# Caffeine-Induced Suppression of GABAergic Inhibition and Calcium-Independent Metaplasticity

**DOI:** 10.1155/2016/1239629

**Published:** 2016-01-14

**Authors:** Masako Isokawa

**Affiliations:** Department of Health and Biomedical Sciences, The University of Texas Rio Grande Valley, One West University Boulevard, Brownsville, TX 78520, USA

## Abstract

GABAergic inhibition plays a critical role in the regulation of neuron excitability; thus, it is subject to modulations by many factors. Recent evidence suggests the elevation of intracellular calcium ([Ca^2+^]_i_) and calcium-dependent signaling molecules underlie the modulations. Caffeine induces a release of calcium from intracellular stores. We tested whether caffeine modulated GABAergic transmission by increasing [Ca^2+^]_i_. A brief local puff-application of caffeine to hippocampal CA1 pyramidal cells transiently suppressed GABAergic inhibitory postsynaptic currents (IPSCs) by 73.2 ± 6.98%. Time course of suppression and the subsequent recovery of IPSCs resembled DSI (depolarization-induced suppression of inhibition), mediated by endogenous cannabinoids that require a [Ca^2+^]_i_ rise. However, unlike DSI, caffeine-induced suppression of IPSCs (CSI) persisted in the absence of a [Ca^2+^]_i_ rise. Intracellular applications of BAPTA and ryanodine (which blocks caffeine-induced calcium release from intracellular stores) failed to prevent the generation of CSI. Surprisingly, ruthenium red, an inhibitor of multiple calcium permeable/release channels including those of stores, induced metaplasticity by amplifying the magnitude of CSI independently of calcium. This metaplasticity was accompanied with the generation of a large inward current. Although ionic basis of this inward current is undetermined, the present result demonstrates that caffeine has a robust Ca^2+^-independent inhibitory action on GABAergic inhibition and causes metaplasticity by opening plasma membrane channels.

## 1. Introduction

Caffeine is a methylxanthine that acts as a nonspecific phosphodiesterase inhibitor [1]. It is widely used as a psychoactive stimulant [2] because it has the ability to interact with neurotransmission and induces a release of excitatory neurotransmitters while blocking adenosine receptors [3]. In addition, caffeine is a structural analogue of strychnine [4]. It competitively binds and antagonizes the glycine receptor. The blockade of glycine receptor by caffeine could synergistically amplify the stimulatory effect of caffeine on excitatory neurotransmission.

Apart from immediate effects on transmitter receptors, caffeine releases calcium from intracellular stores by acting as the agonist of ryanodine receptors [5]. Although the concentration of caffeine that is required to initiate a calcium release is one order higher (which is in a mM range) [6] than the concentration of caffeine acting on adenosine receptors and glycine receptors (which is in a *μ*M range) [7, 8], cafffeine was reported to reduce GABAergic inhibition by initiating a release of calcium from stores and activating calcium-dependent phosphatases that dephosphorylate the GABA_A_ receptor [9]. The requirement of cytosolic calcium was also reported (independently of caffeine) in the regulation of GABA release, mediated by a retrograde messenger called endocannabinoids (eCBs) [10]. In addition to a short-term suppression of GABA release, Ca^2+^-driven eCBs induce long-term depression on GABAergic neuron outputs [11]. Interestingly, the Ca^2+^-dependent and eCB-mediated regulations are limited to only a subpopulation of inhibitory presynaptic terminals. Heterogeneity in the eCB-mediated suppression of GABAergic inhibition suggests complex multilayered arrangements of calcium-dependent modulation of local GABAergic circuits. Since the eCB-mediated GABAergic plasticity involves a calcium release from stores [12, 13], we examined whether caffeine modulated GABAergic inhibition by initiating a calcium release from intracellular stores that leads to the production of calcium-dependent signaling molecules such as eCB.

## 2. Materials and Methods

### 2.1. Preparation of Hippocampal Slices

Hippocampal slice culture was prepared from P6 rat pups according to the method introduced by Stoppini [14]. Animals were decapitated based on the protocol approved by the University of Texas Rio Grande Valley Institutional Animal Care and Use Committee (IACUC) in accordance with the National Institute of Health Guide for the Care and Use of Laboratory Animals (NIH Publications number 80-23). Adequate measures were taken to minimize pain or discomfort. The brain was removed; the hippocampus was dissected from both hemispheres, sliced into 400 *μ*m thick, and placed on a membrane-insert for culturing [15]. For experiments, slices were transferred to a recording chamber and perfused with artificial cerebrospinal fluid (ACSF) consisting of (in mM) 124 NaCl, 3 KCl, 20 glucose, 2 Mg_2_SO_4_, 1.25 NaH_2_PO_4_, 25 NaHCO_3_, and 2 CaCl_2_, while constantly being oxygenated. CA1 pyramidal cells were visualized in slices for electrophysiological recording and optical imaging.

### 2.2. Whole-Cell Recording

Patch pipettes were filled with a solution consisting of (in mM): 110 cesium methanesulphonate, 10 Hepes, 50 CsCl, 1 CaCl_2_, 1 MgCl_2_, 5 QX-314, and 2 MgATP (all from Sigma Chemicals, St. Luis, MO). Fura-2 (100 *μ*M), fura-FF (250 *μ*M), or fluo-4FF (250 *μ*M) was added for Ca imaging (all from Molecular Probes/Life Technologies, Grand Island, NY). Pipette resistance was ~5 MΩ when measured in the bath solution. Tight-seal whole-cell recording was obtained. Series resistance compensation was used to improve the voltage-clamp control (65–85%) (Axopatch 200A, Axon Instruments, Foster City, CA). When access resistance changed more than 15%, data acquisition was stopped, and the cell was discarded from further experimentation. pClamp 10 was used for data acquisition and analysis.

### 2.3. Assessment of Caffeine-Induced Suppression of Inhibition

CA1 pyramidal cells were visually identified and voltage-clamped at −70 mV in the whole cell configuration. A field stimulating electrode (concentric stainless steel, 100 *μ*m in diameter) was placed in the stratum radiatum or stratum oriens. Extracellular ACSF contained 10 *μ*M NBQX and 100 *μ*M D-APV to block ionotropic glutamatergic EPSCs, allowing extracellular stimulation to produce monosynaptic IPSCs. Inhibitory postsynaptic currents (IPSCs) were evoked every 5 s. Caffeine (100 *μ*M to 100 mM, dissolved in ACSF by reducing an equimolar sodium) was applied as a brief local puff application for 1.5 s from a micropipette (2 *μ*m in tip diameter) using Picospritzer (General valve/Parker Hannifin, NJ) while the membrane potential was clamped at a holding potential of −70 mV. For control, regular ACSF was puffed onto the cell. A caffeine puff was applied every 3–5 min. The magnitude of caffeine-induced suppression of IPSCs (%CSI) was determined as follows:

(1) $$\begin{array}{c} \%\text{CSI}=\frac{\left(\text{mean amp. of }5\text{ eIPSC before the caff puff}\right)-\left(\text{mean amp. of }8\text{ eIPSC after the caff puff}\right)}{\text{mean amp. of }5\text{ eIPSC before the caff puff}}\times 100. \end{array}$$

### 2.4. Calcium Imaging

After establishing a whole-cell recording, the cells were held at −70 mV for 10 min before imaging in order for dyes to be diffused and equilibrated in the cytosol. Ca^2+^ signals were acquired from pyramidal cell soma and dendrites using a cooled CCD camera (Photometrics, Tuscan, AR) and IPLab software (Scanalytics/BD Sciences, San Jose, CA) with the sampling rate of 5–10 images/sec for the duration of 20 s. In the case of ratiometric measurements, isosbestic ratioing (380/360) was used. For nonratiometric dyes, a relative increase in fluorescent intensity (Δ*F*/*F*) was calculated. Background was selected from a region away from the cell(s) that were imaged in the same frame and subtracted from the image of interest. Bleaching factor was determined both in cuvette and in cell by illuminating the indicator(s) with the same intensity, duration, and frequency of exposure to that used in the experiments, but without any experimental manipulations. Temporal changes in [Ca^2+^]_i_ in response to caffeine were plotted against time.

### 2.5. Calibration of Calcium Indicators

Ratiometric Ca^2+^ indicators were calibrated according to Grynkiewicz et al. [16] to estimate [Ca^2+^]_i_ (Kd of 131 nM for fura-2 and 5.5 *μ*M for fura-FF, based on the information provided by Molecular Probes). However, Kd measured in buffered solution (reported by Molecular Probes) could be different when the same dye was introduced to the cytosol of intact cells. In our experiments, *R*_min_ and *R*_max_ were measured in situ as follows: CA1 pyramidal cells were whole-cell patch clamped with a pipette that contained 2 mM BAPTA and fura-2 or fura-FF in the recording pipette solution. Five pairs of 380/360 measurements were taken 5 min and 10 min after the break-in. Extracellular ACSF was then switched to a nominally Ca^2+^-free ACSF. The replacement of the extracellular ACSF was checked by the disappearance of eIPSCs (evoked every 3 s). When eIPSCs became undetectable, which occurred in 15–20 min, an additional 5 pairs of 380/360 measurements were taken. These last pairs were averaged and used to calculate *R*_min_. Subsequently, 20 mM CaCl plus 100 *μ*M ionomycin was pressure-ejected for 120 s from a micropipette (2 *μ*m in diameter) that was placed close to the soma. Measurements of fluorescence at 380 and 360 nm were made continuously during the entire ejection period. The 380/360 ratio decreased to 10–16% of the original values within 30 s and remained at that value until the end of the ejection. *R*_max_ was calculated from the lowest ratio value observed. The ratio recovered to 42–50% of the original within 25–30 s after the ejection ended, showing that the decrease was attributable to the rapid perfusion of cell interior with a high [Ca^2+^]_i_ instead of the loss of recording.

## 3. Results

### 3.1. Caffeine Induced Suppression of GABAergic Inhibition

**Figure 1:**
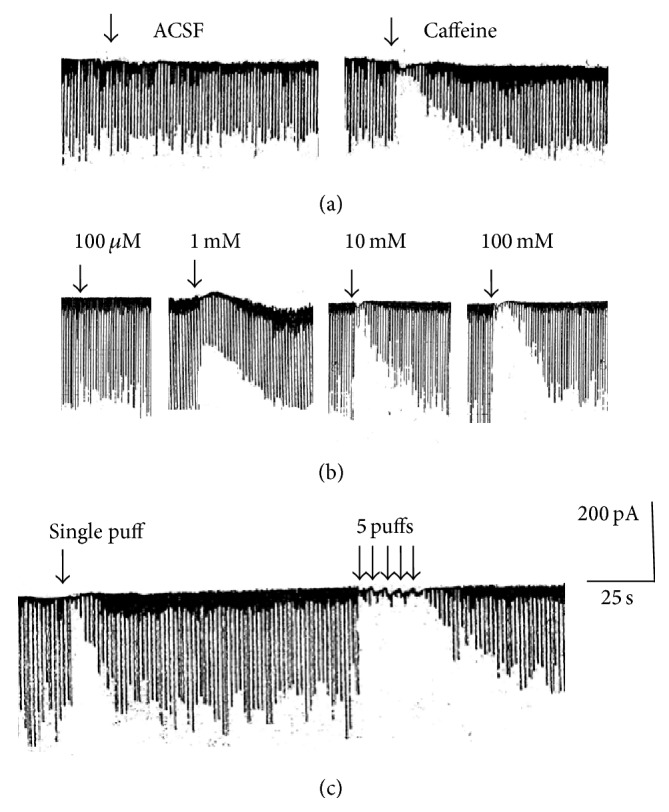
Caffeine-induced suppression of GABAergic IPSC (CSI). No CSI in response to ACSF puff (left trace in a) and a robust CSI in response to caffeine (10 mM) (right trace in a). CSI in response to ascending concentrations of caffeine (b). CSI in response to single caffeine puff application versus repeated application of caffeine puffs (c).

GABAergic IPSCs were isolated in the presence of glutamate receptor antagonists, NBQX (10 *μ*M) and APV (100 *μ*M), while stimulating the stratum radiatum at 0.2 Hz. Caffeine (100 *μ*M–100 mM) was pressure-ejected for 1.5 s from a glass pipette positioned immediately above the recording neuron. Caffeine induced robust instantaneous suppression of IPSCs. The recovery of IPSCs was immediate upon the termination of caffeine application, suggesting that caffeine directly interacted with GABAergic synapses without involving a series of intermediary molecules (Figure 1(a) right trace). In contrast, a control ejection with regular ACSF did not cause any change in the amplitude of IPSCs (Figure 1(a) left trace). We examined whether the magnitude of caffeine-induced suppression of IPSCs (CSI) showed any correlation to the concentration of caffeine. We did not observe any CSI with 100 *μ*M (Figure 1(b)). However, above 1 mM of concentrations, the magnitude of CSI appeared increased in response to ascending concentrations of caffeine (Figure 1(b)). However, we could not quantify CSI to establish a “dose-response” curve because the magnitude of CSI varied among cells in response to a given concentration of caffeine. This was in part due to the difficulty of determining an exact concentration of caffeine at the cell surface after being ejected from the pipette. Although we tried to keep the distance minimum between the pipette tip and the recording cell surface in every recording, a slight change in the distance could cause a variation in the caffeine concentration (caffeine was ejected gently to surrounding ACSF that was constantly perfused at the rate of 2 mL/min).

Repeated application of caffeine puffs (1.5 s/puff × 5 puffs every 5 s) completely blocked IPSCs during the application (Figure 1(c)). The recovery of IPSC amplitude after the repeated application was slower when compared with a single puff application. During repeated application of 5 puffs, we observed the corresponding number of inward currents generated in response to each puff (shown with 5 arrows in Figure 1(c)).

**Figure 2:**
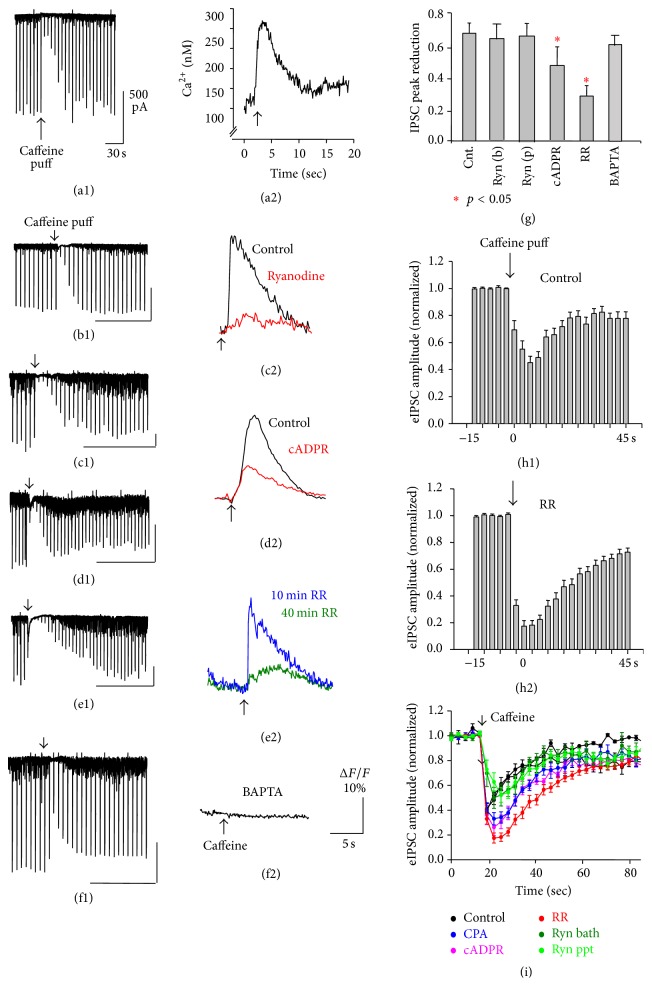
Caffeine-induced suppression of GABAergic IPSCs (CSI) and concomitant increase in cytosolic calicum. Both measurements were recorded simultaneously from the same cell during the whole cell patch clamp recording. (a1 and b1) in control ACSF, (c1) in ryanodine in pipette, (d1) in cADPR in pipette, (e1) in ruthenium red (RR) in pipette, and (f1) with BAPTA (20 mM) in pipette. Caffeine was applied at the arrow. Reduction in IPSC peak amplitude is summarized in (g). Time-dependent changes in IPSC amplitude in control (h1) and ruthenium red (h2) (15 neurons each). Magnitude of CSI in response to control, CPA (cyclopiazonic acid, 30 *μ*M), cADPR (10 *μ*M), ruthenium red (20 *μ*M), and ryanodine in pipette (100 *μ*M) and in the bath (20 *μ*M) (i). Calibrations in (b1, c1, d1, e1, and f1): 500 pA, 60 s. Calibration in (f2) is shared by (c2, d2, and e2).

CSI was accompanied with an increase of cytosolic calcium to 300 nM when measured with an intracellular application of fura-2 (Figure 2(a2)). Although this measurement indicated an estimated calcium concentration generated by caffeine during CSI, we should be careful of determining a cytosolic calcium level because (1) the comparability to physiological conditions is always difficult due to the effect that bicarbonate has on intracellular calcium concentration and (2) washout effects (by the whole cell approach) exist [17].

We applied ryanodine (100 *μ*M), cADPR (100 *μ*M), and ruthenium red (10 *μ*M) intracellularly by dissolving these compounds in the recording pipette solution. cADPR is an agonist of the ryanodine receptor and facilitates a release of calcium from ryanodine-sensitive stores to empty them. Inclusion of cADPR in the recording pipette exhibited a decrease in [Ca^2+^]_i_ in response to caffeine in 15 min after break-in (Figure 2(d2) where control shows a calcium increase in response to caffeine at the time of break-in). Ryanodine and ruthenium red both block the release of calcium from stores. They decreased [Ca^2+^]_i_ in response to caffeine in 15 min after break-in (Figures 2(c2) and 2(e2) where control shows a calcium increase in response to caffeine at the time of break-in). In addition, BAPTA (10 mM) was applied intracellularly from a recording pipette, which completely clamped the concentration of cytosolic calcium during caffeine application (Figure 2(f2)). Under these conditions, caffeine-induced suppression of IPSCs (CSI) persisted in spite of the absence of intracellular-calcium rise (Figures 2(b1)–2(f1)). In particular, the magnitude of CSI was greatest in the presence of ruthenium red, an inhibitor of the ryanodine receptor (Figures 2(e1), 2(g), and 2(h2)). The magnitude of CSI, caused by caffeine in the presence of these compounds, is summarized in Figure 2(i).

### 3.2. Caffeine Induced Inward Currents

**Figure 3:**
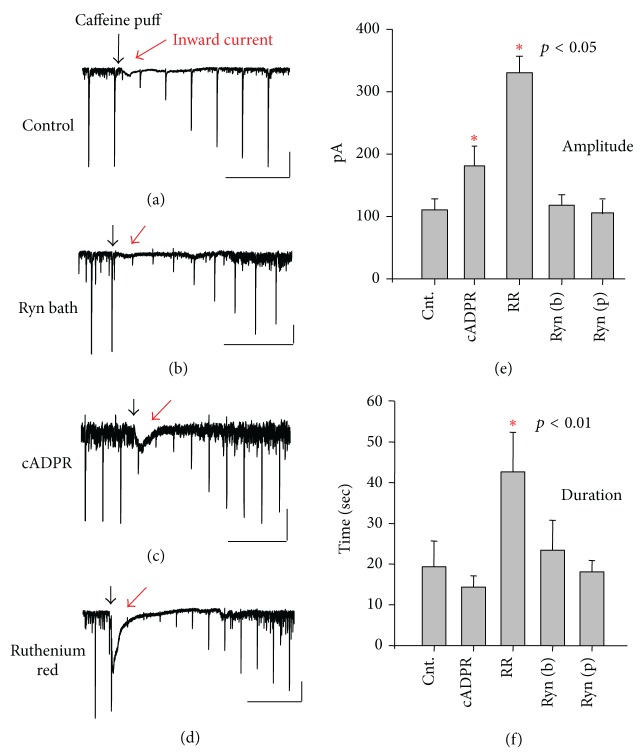
Caffeine-induced inward current. Local puff application of caffeine induced an inward current in control ACSF (red arrow in a) and in the presence of agonist and antagonist of the ryanodine receptor (b, c, and d). Calibrations: 50 pA and 20 s for (a and b); 200 pA and 20 s for (c); and 100 pA and 20 s for (d). Amplitude (e) and duration (f) of caffeine-induced inward currents in response to intracellular application of cADPR, ruthenium red (RR), and ryanodine.

We observed the generation of an inward current in response to a local brief puff application of caffeine. The inward current was present with a moderate magnitude in control (Figure 3(a)). The intrapipette application of ryanodine inhibited the amplitude of the inward current (Figure 3(b)) and cADPR increased the amplitude of the inward current (Figure 3(c)). This suggests the possibility that the inward current was a result of calcium release from stores; thus possibly opened store-operated channels. However, contrary to the above interpretation, ruthenium red, which inhibits the ryanodine receptor and blocks a release of calcium from stores (Kd ~ 20 nM, [18]), accentuated the magnitude of the inward current (Figure 3(d)). Indeed, the inward current became maximum 40 min after the introduction of ruthenium red via a recording pipette. The amplitude of inward current (Figure 3(e)) and the duration (Figure 3(f)) in response to cADPR, ryanodine, and ruthenium red, are summarized in 13 neurons in 11 hippocampi.

## 4. Discussion

The present study demonstrates the ability of caffeine to interfere GABAergic inhibition independent of the rise in concentration of intracellular calcium. The inhibitory action of caffeine was rapid on GABAergic IPSCs suggesting that the effect of caffeine was direct postsynaptically on the GABA receptor and/or presynaptically at the GABA release site, independently of calcium. The present study also demonstrates the generation of inward currents during the blockade of GABAergic IPSCs by a topical application of caffeine. The inward current amplitude changed in response to the agonist and antagonists of the ryanodine receptor and showed metaplasticity in the presence of ruthenium red independently of calcium.

Caffeine increases cAMP and cGMP by inhibiting phosphodiesterase. cGMP modulates neurotransmitter release from presynaptic axon terminals, including GABA, through the activation of protein kinase G (PKG) [19]. In addition, cAMP and cGMP open the cyclic nucleotide-gated channels (CNG) [20], which is highly expressed in soma and proximal dendrites of central neurons including the hippocampus [21]. Thus, caffeine may inhibit GABA release by activating cyclic nucleotide-gated Ca^2+^-permeable channels. On the other hand, there are reports to show that caffeine potentiated the release of GABA by initiating a calcium release from caffeine-ryanodine-sensitive stores [22] and by activating the NMDA receptor and the A1 adenosine receptor [7]. Additional experiments on paired-pulse ratio and the frequency analysis of spontaneous IPSCs will help identify a possible CSI expression site in the present study.

Postsynaptically, independently of [Ca^2+^]_i_, caffeine competitively binds to multiple regulatory sites of the GABA_A_ receptor and interferes with GABAergic transmission [1]. Caffeine also disrupts chloride transporters and shifts the chloride equilibrium potential towards the reduction of its conductance [23]. Taketo and coworkers [24] reported the inhibition of GABAergic IPSCs by caffeine independently of intracellular calcium mobilization by bath-applying caffeine over several minutes (instead of 1.5 s local puff ejection, which was used in the present study) in CA3 pyramidal cells. They found that the amplitude of IPSCs was reduced by caffeine and the reduction was insensitive to intracellular application of ryanodine, ruthenium red, and BAPTA. This is in agreement with the results reported in the present study. Taketo and coworkers also reported that the caffeine's effect was not mediated by the adenosine receptor, cAMP, or PKA. Although they did not specify the mechanism by which caffeine inhibited the IPSC, they suggested that a network-driven and calcium-dependent modulation of the IPSC might occur in some brain region after caffeine application and concluded that their results did not necessarily exclude a possible contribution of [Ca^2+^]_i_ in modulation of GABAergic IPSCs. Multiple actions of caffeine on GABAergic synapses and homeostasis suggest caffeine's powerful “affinity” for successful access to fundamental mechanisms in inhibitory neurotransmission.

We acknowledge that our findings on the caffeine-induced inward current and its amplification in the presence of ruthenium red are preliminary. Generation of inward currents by a brief topical application of caffeine has not so far been established. In the available pool of literatures, caffeine is suggested to interact with various types of K^+^ currents including Ca^2+^-activated K^+^ currents (BK, SK, and IK), inwardly rectifying K^+^ current, M-current, and the Ca^2+^-activated Cl^−^ current. These currents might directly or indirectly influence neuronal membrane resistance and thus modulate cell's excitability, which could affect GABAergic inhibitory transmission. Caffeine-induced inward current that we observed in the present study may be similar to the inward current revealed as a consequence of the blockade of M-current by muscarine (muscarine-sensitive K^+^ current) [25]. On the other hand, the inward current induced by caffeine could be a nonspecific cation current with high permeability to Ca^2+^ such as Ca^2+^-release activated Ca^2+^ entry (CRAC). Ruthenium red is known to interact with various calcium-permeable channels and transporters including ryanodine receptors (RyR1, RyR2, and RyR3), TRIP channels (TRPM6, TRPM8, TRPV1, TRPV2, TRPV3, TRPV4, TRPV5, TRPV6, TRPA1, and TRPP3), calcium homeostasis modulator 1 (CALHM1), calcium pumps (Ca^2+^-ATPase), mitochondrial Ca^2+^ uniporter, and Ca^2+^ binding proteins including calmodulin [26–28]. Further investigation on the identification of (1) ion channels that are activated by caffeine and (2) the expression site of CSI would improve elucidation of the mechanism and unidentified role of caffeine in the regulation of GABAergic inhibition.
